# The effects of anesthesia induction and positive pressure ventilation on right-ventricular function: an echocardiography-based prospective observational study

**DOI:** 10.1186/s12871-019-0870-z

**Published:** 2019-11-04

**Authors:** Harry Magunia, Anne Jordanow, Marius Keller, Peter Rosenberger, Martina Nowak-Machen

**Affiliations:** 1 Department of Anaesthesiology and Intensive Care Medicine, University Hospital Tübingen, Eberhard-Karls-University Tübingen, Hoppe-Seyler-Str. 3, 72076 Tübingen, Germany; 2 Department of Anesthesia and Intensive Care Medicine, Klinikum Ingolstadt, Krumenauerstr. 25, 85049 Ingolstadt, Germany

**Keywords:** Right ventricular function, Positive-pressure respiration, Anesthesiology, Echocardiography, Three-dimensional

## Abstract

**Background:**

General anesthesia induction with the initiation of positive pressure ventilation creates a vulnerable phase for patients. The impact of positive intrathoracic pressure on cardiac performance has been studied but remains controversial. 3D echocardiography is a valid and MRI-validated bed-side tool to evaluate the right ventricle (RV). The aim of this study was to assess the impact of anesthesia induction (using midazolam, sufentanil and rocuronium, followed by sevoflurane) with positive pressure ventilation (PEEP 5, tidal volume 6–8 ml/kg) on 2D and 3D echocardiography derived parameters of RV function.

**Methods:**

A prospective observational study on fifty-three patients undergoing elective cardiac surgery in a tertiary care university hospital was designed. Transthoracic echocardiography exams were performed before and immediately after anesthesia induction and were recorded together with hemodynamic parameters and ventilator settings.

**Results:**

After anesthesia induction TAPSE (mean difference − 1.6 mm (95% CI − 2.6 mm to − 0.7 mm; *p* = 0.0013) as well as the Tissue Doppler derived tricuspid annulus peak velocity (TDITVs’) were significantly reduced (mean difference − 1.9% (95% CI: − 2.6 to − 1.2; *p* < 0.0001), but global right ventricular ejection fraction (RVEF; *p* = 0.1607) and right ventricular stroke volume (RVSV; *p* = 0.1838) did not change.

**Conclusions:**

This data shows a preserved right ventricular ejection fraction and right ventricular stroke volume after anesthesia induction and initiation of positive pressure ventilation. However, the baso-apical right ventricular function is significantly reduced. Larger studies are needed in order to determine the clinical impact of these findings especially in patients presenting with impaired right ventricular function before anesthesia induction.

**Trial registration:**

Retrospecitvely registered, 6th June 2016, ClinicalTrials.gov Identifier NCT02820727.

## Background

Anesthesia induction and initiation of intermittent positive pressure ventilation (IPPV) can lead to hemodynamic impairment due to several factors. IPPV is a key element of general anesthesia that is routinely used for major surgical procedures and as part of intensive care treatment provided to critically ill patients. The impact of positive intrathoracic pressure during IPPV and positive end expiratory pressure (PEEP) on the circulatory system has been studied and well described [1, 2]. The pathophysiological effects of IPPV with PEEP mainly consist of decreased venous return, decreased right ventricular (RV) output with a potential increase in RV end diastolic volume, increase in RV afterload and decreased pulmonary blood flow, which can disturb hemodynamic status in certain patients [3]. In addition, all anesthetic agents used for the induction and maintenance of general anesthesia have varying degrees of impact on systemic vascular resistance and cardiac contractility and can, hence, potentially impair right ventricular function [4]. However, the controversy of whether an increase in intrathoracic pressure during IPPV actually leads to an decreased right ventricular function and ejection fraction remains unclear, as some studies did not find a decrease in right ventricular filling pressures or RV function after the application of high PEEP levels [5, 6]. Other factors that contribute to hemodynamic changes during anesthesia induction are therapeutic measures, e.g. fluid administration and vasopressor therapy.

Two-dimensional (2D) transthoracic echocardiography has been used as a standard imaging modality to evaluate both cardiac dimensions and functions as well as inferior vena cava (IVC) diameters during varying levels of intrathoracic pressure in different clinical settings [7, 8]. Due to the complex anatomical shape and functional complexity of the right ventricle, 2D-echocardiography still relies on multiple surrogate parameters [9, 10]. Surrogate parameters used to estimate RV function are the tricuspid annular plane systolic excursion (TAPSE), the right ventricular fractional area change (RVFAC) or the tissue Doppler derived systolic movement of the lateral tricuspid annulus (TDITVs’) [11]. In recent years, real-time 3-dimensional echocardiography (3DE) has become increasingly available as a bedside tool for clinicians worldwide [12, 13]. 3DE has been thoroughly validated against cardiac magnetic resonance imaging (CMRI) and offers the possibility to quantitatively assess right ventricular function and filling states based on volumetric models calculated from 3-dimensional volume datasets [14, 15]. This overcomes the problem of using surrogates while evaluating RV function.

To the best of our knowledge, this is the first study explicitly examining the effects of anesthesia induction and positive pressure ventilation on right ventricular function using 3D echocardiography. The specific aim of this prospective observational study was to examine the effects of anesthesia induction and IPPV on conventional 2D-echocardiography and 3D-echocardiography-derived determinants of right ventricular function in patients scheduled for cardiac surgery.

## Methods

### Study design

Approval of this prospective study (ClinicalTrials.gov Identifier NCT02820727) was given by the Institutional Review Board at the University Hospital Tübingen (IRB# 320/2015BO2). Sample size was calculated for comparing paired differences. The calculated sample size to achieve a power of 80% and a level of significance of 0.05 (two tailed), for detecting a 2 ± 5 mm change in TAPSE (effect size of 0.4) was *n* = 52. During the study period from 11/2015 to 11/2016, 554 adult patients above the age of 18 years who were scheduled for elective cardiac surgery at a tertiary care university hospital were screened for possible inclusion. The inclusion criteria were sinus rhythm and ability to give informed consent. Patients were only asked to give informed consent if an investigator capable of performing a comprehensive 3DE was available on the day of surgery. Right ventricular function was assessed by real time three-dimensional transthoracic echocardiography (TTE) immediately before anesthesia induction under spontaneous ventilation and as soon as possible after achieving hemodynamic stability after anesthesia induction and endotracheal intubation under controlled IPPV with constant PEEP. The study was performed according to the STROBE statement for observational studies [16].

### Anesthesia management

Standardized monitoring was applied in all patients. The patients were not premedicated. All patients received an arterial cannula for blood pressure monitoring before anesthesia induction. Midazolam (0.1–0.15 mg/kg), sufentanil (0.3–0.5 μg/kg) and rocuronium (0.5–1 mg/kg) were applied intravenously (i.v.) for anesthesia induction and endotracheal intubation and were followed by sevoflurane (up to 1 minimal alveolar concentration (MAC)) for anesthesia maintenance. As per institutional standard, intravenous volume was administered restrictively using isotonic crystalloid solutions with a maximum of 15 ml/kg prior to initiation of cardiopulmonary bypass in all patients. A target mean arterial blood pressure (MBP) of > 65 mmHg was maintained during anesthesia induction using a continuous i.v. infusion of noradrenaline as a first line agent to counteract hypotension based on the clinical requirements. Pressure controlled positive pressure ventilation with a standardized PEEP of 5 cm H_2_O and a peak inspiratory pressure (Pinsp) to receive a tidal volume of 6-8 ml/kg was initiated, and the inspiratory oxygen fraction was titrated to individual demands to maintain a SpO2 between 95 and 99%. End-tidal carbon dioxide levels were maintained between 35 and 37 mmHg by adjusting the ventilation frequency. All hemodynamic parameters were recorded during the echocardiography studies.

### Echocardiography

**Fig. 1:**
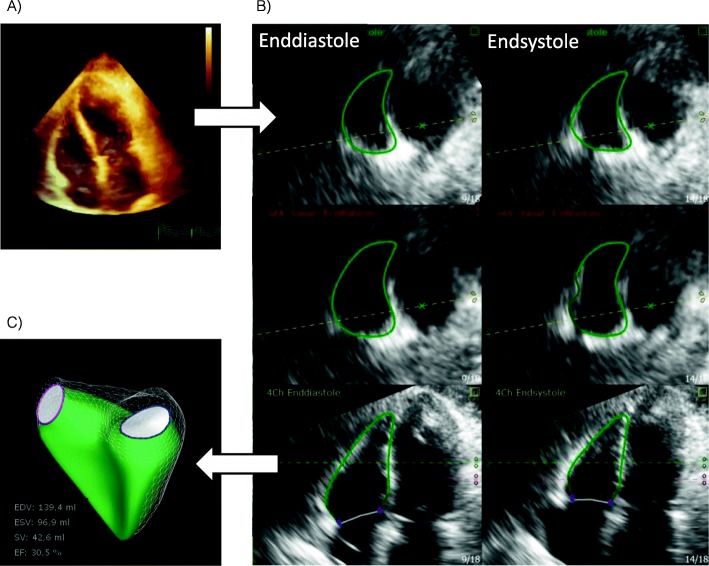
**a** 3D full volume dataset of the left and right ventricle based on an apical 4-chamber view. **b** Endocardial border tracking by the Tomtec 4D RV-function package. The first column shows the enddiastolic frame, the second column shows the endsystolic frame. The First and second lines show medial and basal transversal planes of the RV. The third line shows the right ventricle in a reconstructed apical view. **c** Results of the Tomtec 4D RV-function package showing the volumetric model of the RV. The mesh grid shows the enddiastolic volume, the green inner model shows the endsystolic volume. Absolute values of volumes and the ejection fraction are also reported

A transthoracic real time 3DE study (Philips iE33-system, X5–1 Matrix transducer, Philips Healthcare Inc., Andover, USA) was performed in all patients by two experienced echocardiographers, trained in performing 3D imaging of the right ventricle. Echocardiography loops were recorded before and after anesthesia induction from transapical windows. The study protocol consisted of a 2D transapical 4-chamber view and 3D full volume acquisitions including the whole right and left ventricle. The 3D full volume datasets were acquired using an ECG-gated 4 heart beats-technique with a short apnoeic phase maintaining PEEP. Hemodynamic parameters were allowed to stabilize prior to the TTE exam. Tricuspid annular plane systolic excursion (TAPSE) was measured in an apical 4-chamber view using M-mode on the lateral tricuspid annulus. Systolic apical movement velocity of the lateral tricuspid annulus (TDITVs’) was assessed using tissue Doppler imaging. All measurements were performed according to current ASE recommendations [11]. All 3D quantifications were performed offline. RV quantification was performed on a vendor independent platform (Tomtec Image Arena; Tomtec Imaging Systems GmbH, Unterschleissheim, Germany) by two trained echocardiographers blinded for each other’s results. For quantification of the 3D datasets, software that had been validated against cardiac magnetic resonance imaging (CMRI) was used (Tomtec 4D RV-Function 2.0; Tomtec Imaging Systems GmbH, Unterschleissheim, Germany) [14, 15]. After adjustment of the 3D image in pre-defined axes, this software uses a speckle tracking based technique to identify right ventricular endocardial borders to generate a dynamic model of the right ventricular endocardial surface (Fig. 1). The RV end-diastolic (RVEDV) and end-systolic (RVESV) volumes were measured based on the generated RV model. RVEF was calculated as [(RVEDV-RVESV)/RVEDV] × 100%. To compare individual RV sizes, volumes were indexed to the body-surface area (RVEDVI and RVESVI). Based on the 3D RV model, the right ventricular free wall peak longitudinal strain (3D-RVLS-fw) and right ventricular septal-wall peak longitudinal strain (3D-RVLS-sw) was assessed as the difference in the measured distance of the curvature in systole and diastole of the free or septal RV wall: [(RV end-diastolic curvature (mm)-RV end-systolic curvature (mm))/RV end-diastolic curvature (mm))] × 100%. Stroke volume (SV) was calculated as SV = RVEDV * RVEF [17].

### Statistical analysis

The normal distribution of the variables was assessed using skewness, kurtosis and the Shapiro-Wilk’s tests. Continuous variables are reported as the mean values ± standard deviation. For echocardiographic and hemodynamic parameters, 95% confidence intervals of the mean are reported. Comparison of continuous variables between the different groups was performed using a Student’s t test. Variables that were not normally distributed are reported as median and interquartile ranges (IQR). Comparison of these variables was performed using the Wilcoxon Rank-Sum test. The values of the different time points were compared using a two-tailed paired T-test. Categorical variables are reported as percentages. Differences in the variables were interpreted as significant at *p* values < 0.05.

To assess intra-observer reliability, 10 randomly selected datasets were analyzed by the initial investigator, blinded to the initial results, at a different time point. To calculate inter-observer reliability, 10 randomly selected datasets were assessed by a second investigator blinded for the results of the first investigator. Intraclass correlation coefficients (ICC) and corresponding 95% confidence intervals were calculated to assess intra-observer and inter-observer reliability.

All statistical analyses were performed using the SAS JMP software (ver. 14.0.0, SAS Institute Inc., Cary, USA) and IBM SPSS statistics (ver. 24, IBM, Armonk, USA).

## Results

Fifty-nine patients gave informed consent to take part in this study. Three patients could not be studied due to changes in surgical planning, and three patients had a low imaging quality (stitching artefacts) insufficient for 3D volumetry. Fifty-three patients (44 male and 9 female) were included into the final analysis.

### Patient characteristics

The median age of the patients was 64 years (IQR 55 to 75) having a median EuroSCORE II of 2.9% (IQR 1.9 to 7.5). Further demographic data as weight, height, body-surface-area, body-mass-index (BMI), preoperative laboratory values (creatinine, quick, total bilirubin) and the planned cardiac surgeries are given in Table 1.

**Table 1:** Demographics, laboratory values, anesthesia induction agents and ventilator settings

Demographics and laboratory values	
Age [years]	64 (IQR 55 to 75)
Gender	
Male	44
Female	9
BMI [kg/m^2^]	27 (SD 4)
BSA [m^2^]	1.97 (SD 0.2)
Weight [kg]	82.5 (SD 14.8)
Height [cm]	174.5 (SD 8)
Planned cardiac surgeries	
CABG	16
AVR	13
MVR	6
Combination	15
Other	3
EuroSCORE II [%]	2.9 (IQR 1.9 to 7.5)
Creatinine [mg/dl]	0.9 (SD 0.2)
Prothrombin time (Quick’s value) [%]	95 (SD 22)
Total Bilirubin [md/dl]	0.7 (IQR 0.5 to 0.9)
Anaesthesia induction agents and noradrenaline doses	
Midazolam [mg/kg]	0.14 (IQR 0.11 to 0.16)
Sufentanil [μg/kg]	0.56 (IQR 0.47 to 0.59)
Rocuronium [mg/kg]	0.79 (SD 0.26)
Noradrenaline Dose [μg/kg/min]	0.02 (IQR 0 to 0.05)
Parameters of pressure-controlled ventilation	
PEEP Level [mbar]	5
P insp [mbar]	15 (IQR 14 to 17)
TV [ml/kg]	7 (IQR 6 to 8)

*BSA* body-surface area, *BMI* body-mass index, *CABG* coronary artery bypass grafting, *AVR* aortic valve repair/replacement, *MVR* mitral valve repair/replacement, *PEEP* positive end-expiratory pressure, *P insp* inspiratory peak pressure, *TV* tidal volume

### Anesthesia induction and ventilator settings

The median level of the PEEP was 5 mbar, the median Pinsp was 15 (IQR 14 to 17) to obtain a TV of 7 ml/kg (IQR 6 to 8). Anesthesia induction agents are given in Table 1. No patient received noradrenaline prior to anesthesia. Median noradrenaline dose after anesthesia induction was 0.02 μg/kg/min (IQR 0 μg/kg/min to 0.05 μg/kg/min). During anesthesia induction the patients received a median dose of 330 ml (IQR 250 ml to 440 ml) of crystalloid fluids.

### Hemodynamic data

Hemodynamic variables are given in Table 2. Patients experienced a significant reduction in heart rate (75 (SD 15) bpm to 66 (SD 14) bpm, *p* = < 0.0001), systolic blood pressure (SBP; 136 (SD 24) mmHg to 112 (SD 19) mmHg; *p* < 0.0001), diastolic blood pressure (DBP; 70 (IQR 60 to 80) mmHg to 57 (SD 11) mmHg; *p* < 0.0001) and mean blood pressure (MBP; 91 (IQR 80 to 104) mmHg to 78 (SD 12) mmHg; *p* < 0.0001).

**Table 2:** Hemodynamic and echocardiographic parameters

	Pre	Post	Mean Difference (95% CI)	*p* value
Hemodynamic parameters				
HR [bpm]	75 (SD 15)	66 (SD 14)	−9 (−12 to −5)	< 0.0001
Systolic BP [mmHg]	136 (SD 24)	112 (SD 19)	−24 (−32 to − 17)	< 0.0001
Diastolic BP [mmHg]	70 (IQR 60 to 80)	57 (SD 11)	−13 (− 18 to −8)	< 0.0001 ^a^
Mean BP [mmHg]	91 (IQR 80 to 104)	75 (SD 12)	−17 (− 22 to − 12)	< 0.0001 ^a^
Echocardiographic parameters				
Right ventricle				
RVEDAI (cm^2^/m^2^) *n* = 49	13 (SD 3)	13 (SD 2)	−0.3 (−1 to 0.4)	0.3585
RVEDV (ml) *n* = 53	132 (SD 35)	132 (SD 36)	−0.0 (−8 to 8)	0.9996
RVESV (ml) *n* = 53	76 (SD 25)	78 (SD 27)	2.4 (−3.2 to 8.1)	0.3923
RVEDVI (ml/m^2^) *n* = 53	67 (SD 17)	67 (SD 17)	−0.3 (−5.2 to 4.5)	0.9609
RVESVI (ml/m^2^) *n* = 53	35 (IQR 32 to 44)	37 (IQR 30 to 45)	1.1 (−1.8 to 3.9)	0.4482
RVFAC (%) *n* = 49	38 (SD 9)	38 (SD 9)	−0.3 (−2.4 to 1.8)	0.7538
RVEF (%) *n* = 53	43 (SD 9)	42 (SD 8)	−1.4 (− 3.5 to 0.6)	0.1607
Stroke Volume (ml) *n* = 53	57 (SD 19)	54 (SD 16)	−3 (−7.5 to 1.5)	0.1838
TAPSE (mm) *n* = 51	18 (SD 4)	16 (SD 4)	−1.6 (−2.6 to −0.7)	0.0013
TDI TV s’ (cm/s) *n* = 43	13.2 (IQR 11.8 to 15.9)	11.8 (IQR 10.1 to 13.3)	−1.9 (−2.6 to − 1.2)	< 0.0001
3D-RVLS-fw (%) *n* = 52	−22.9 (SD 6)	− 21.4 (SD 5.9)	1.5 (0.1 to 2.9)	0.0366
3D-RVLS-sw (%) *n* = 52	−12.8 (SD 5.2)	− 12.3 (SD 3.9)	−0.3 (− 1.2 to 1.9)	0.5492
Tricuspid valve regurgitation (n=)				
None or trace	26			
Mild	25			
Moderate	2			
Severe	0			
Left ventricle				
LVEDVI (ml/m2) *n* = 38	80 (SD 24)	79 (SD 25)	−0.5 (−4.3 to 3.3)	0.7880
3D-LVEF (%) *n* = 38	53 (SD 16)	52 (SD 13)	−0.9 (−4.1 to 2.3)	0.5704

Pre: baseline values; Post: Post-anesthesia induction values; CI: confidence intervals (lower limit to upper limit); HR: heart rate; BP: blood pressure; RVEDAI: right ventricular end-diastolic area index to body surface area to RVEDVI: right ventricular end-diastolic volume indexed to body surface area to RVFAC: right ventricular fractional area change to 3D-RVEF: right ventricular ejection fraction derived by 3D volumetry to TAPSE: tricuspid plane systolic excursion to TDI TV s’: tissue Doppler derived peak systolic velocity of the lateral tricuspid annulus to 3D-RVLS-fw: right ventricular free wall peak longitudinal strain derived by 3D echocardiography to 3D-RVLS-sw: right ventricular septal wall peak longitudinal strain derived by 3D echocardiography to LVEDVI: left ventricular end-diastolic volume indexed to body surface area by 3D volumetry to LVEF: left ventricular ejection fraction derived by 3D volumetry

^a^Wilcoxon signed-rank test

### Echocardiographic data

**Fig. 2:**
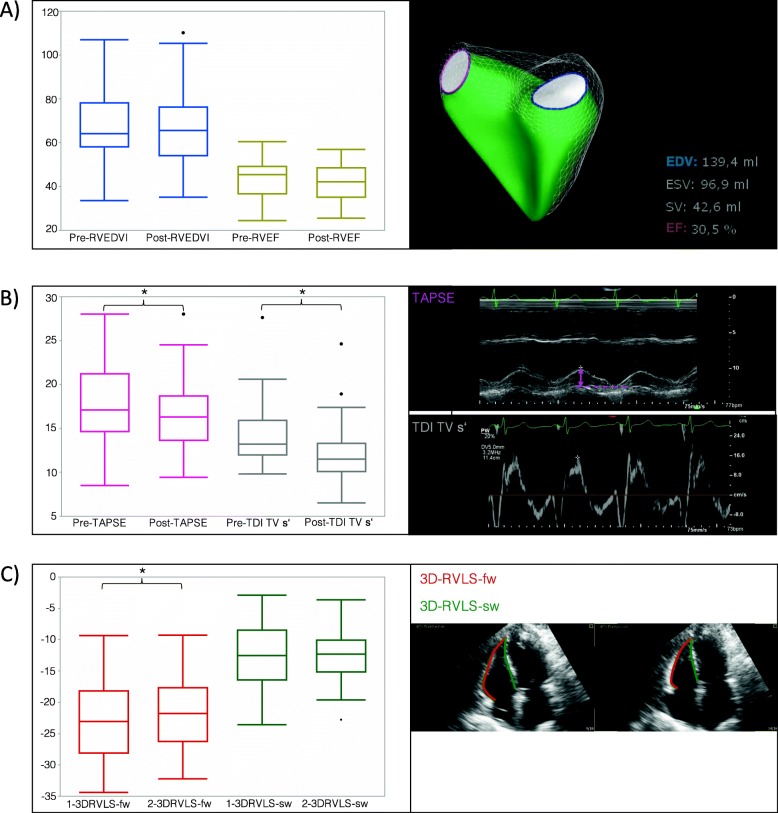
**a** Bar graphs of the right ventricular end-diastolic volume indexed to body surface area (RVEDVI) and right ventricular ejection fraction (RVEF) before and after anesthesia induction are shown. No changes were observed regarding RVEDVI and RVEF. Right side shows an example of a volumetric model of the right ventricle together with an example of results given by the Tomtec 4D RV-function software. **b** Bar graphs of the tricuspid annular plane systolic excursion (TAPSE) and tissue Doppler-derived peak systolic velocity of the lateral tricuspid annulus (TDI TV s’) before and after anesthesia induction are shown. Significant reductions were observed for the TAPSE and TDI TV s’. Right side shows examples of the TAPSE measurement in M-Mode and the measurement of the TDI TV s’ in tissue spectral Doppler. **c** Bar graphs of the right ventricular peak systolic free wall strain (3DRVLS-fw) and septal wall strain (3DRVLS-sw) are shown. A significant decrease in 3DRVLS-fw was observed with no change in 3DRVLS-sw. Right side shows an example of the strain measurements in reconstructed 3D datasets

A summary of the echocardiographic parameters is given in Table 2. No significant change in the RVEDV, RVEDVI, RVESV, RVESVI, RVEF, right ventricular end-diastolic area indexed to body surface area (RVEDAI), RVFAC, 3D-RVLS-sw or right ventricular stroke volume was recorded (Fig. 2 a).

After anesthesia induction and IPPV, the TAPSE was significantly reduced (18 (SD 4) mm to 16 (SD 4) mm; *p* = 0.0013) (Fig. 2 b). The TDITVs’ was also significantly reduced (13.2 cm/s (IQR 12 to 15.9) to 11.8 cm/s (IQR 10.1 to 13.3), *p* < 0.0001). 3D-RVLS-fw also significantly decreased (− 22.9 (SD 6) % to − 21.4 (SD 5.9) %, *p* = 0.0366) (Fig. 2 c).

There was no significant correlation between the noradrenaline dose and the change in RVEF (r = 0.106, *p* = 0.4451), 3D-RVLS-fw (r = − 0.17, *p* = 0.2284), TAPSE (r = − 0.073, *p* = 0.6129) or TDITVs’ (r = 0.07, *p* = 0.6439).

### Subgroup analysis: patients with normal or impaired RVEF

For a subgroup analysis, patients were grouped based on their baseline RVEF. A baseline RVEF above 35% was considered normal, a baseline RVEF below or equal to 35% was considered depressed. Fourty-one patients with a median age of 64 years (IQR 53 to 74) were categorized into the group with a normal RVEF, twelve patients with a median age of 72 (IWR 63 to 77) were categorized into the group with depressed RVEF.

In both groups HR and MAP were significantly lower after anesthesia induction compared to the baseline values. RVEDV did not significantly change in either of the groups. In the group with a normal RVEF, all parameters reflecting RV function (RVEF, TAPSE, TDITVs’ and 3D-RVLS-fw) worsened significantly (Table 3). The group with the depressed RVEF at baseline did not show a significant change of the RV functional parameters.

**Table 3:** Hemodynamic and echocardiographic parameters grouped by RVEF threshold of 35%

	RVEF > 35% *N* = 41				RVEF ≤35% *N* = 12			
Age	64 (IQR 53 to 74)				72 (IQR 63 to 77)			
BMI (kg/m^2^)	26 ± 4				29 ± 4			
EuroSCORE II [%]	2.8 (IQR 1.4 to 5.9)				7 (IQR 2.2 to 16.6)			
Noradrenaline dose [μg/kg/min]	0 (IQR 0 to 0.04)				0.03 (IQR 0 to 0.07)			
Time point	Pre	Post	*p* value	95%CI of the mean difference	Pre	Post	*p* value	95%CI of the mean difference
HR [bpm]	74 ± 15	66 ± 13	0.0005	−8 (−3 to −12)	77 ± 17	66 ± 15	0.0012	−11 (−6 to − 17)
MAP [mmHg]	91 (IQR 78 to 102)	75 ± 13	< 0.0001^a^	−17 (− 11 to −23)	91 (IQR 81 to 109)	78 ± 9	0.0005^a^	− 16 (−7 to − 26)
RVEDV [ml]	130 ± 35	133 ± 34	0.5479	4 (13 to −5)	145 ± 38	132 ± 44	0.1115	−14 (4 to −31)
RVESV [ml]	69 ± 19	75 ± 24	0.0253	7 (12 to 1)	102 ± 26	90 ± 36	0.0807	−12 (2 to −25)
RVEDVI [ml/m^2^]	66 ± 16	68 ± 16	0.5416	2 (7 to −3)	64 (IQR 59 to 82)	65 ± 20	0.0923^a^	−7 (1 to −17)
RVESVI [ml/m^2^]	35 ± 9	38 ± 11	0.0281	3 (6 to 0)	51 ± 14	44 ± 17	0.0594	−6 (0 to −13)
RVEF [%]	47 ± 6	43 (IQR 40 to 49)	0.0063^a^	−3 (0 to −5)	30 (26 to 34)	32 (28 to 37)	0.3394^a^	3 (8 to −2)
RV stroke volume [ml]	61 ± 19	57 ± 16	0.2001	−3 (2 to −9)	37 (IQR 35 to 54)	41 (IQR 32 to 54)	0.6221^a^	−1 (7 to −10)
TAPSE [mm]	19 ± 4	17 ± 4	0.0033 *N* = 39	−2 (−1 to −3)	14 ± 3	14 (IQR 11 to 15)	0.2334^a^	−1 (1 to −3)
TDI TV s‘[cm/s]	14.5 ± 3.3 *N* = 33	12 ± 3.7	< 0.0001 *N* = 33	−2.3 (−1.5 to −3.1)	13.3 ± 3.5	11.6 (IQR 10.5 to 13.5)	0.5371^a^	−1 (1 to − 2)
3D-RVLS-fw [%]	−25 ± 4.9	− 22.9 ± 5.9	0.0143	2 (3.5 to 0.4)	−16 ± 3.2	−17.7 (IQR − 20.8 to − 10.5)	1.0000^a^	0 (3.3 to − 3.5)
3D-RVLS-sw [%]	−13.7 ± 5.3	− 13 ± 3.7	0.5709	0.5 (2.3 to − 1.3)	− 9.7 ± 3.5	−9.4 ± 3.3	0.8490	0.3 (3.6 to − 3)

Pre: baseline values; Post: post-anesthesia induction values; RVEDV: right ventricular end-diastolic volume; RVESV: right ventricular end-systolic volume; RVEDVI: right ventricular end-diastolic volume indexed to body surface area; RVESVI: right ventricular end-systolic volume indexed to body surface area; 3D-RVEF: right ventricular ejection fraction derived by 3D volumetry; TAPSE: tricuspid plane systolic excursion; TDI TV s’: tissue Doppler derived peak systolic velocity of the lateral tricuspid annulus; 3D-RVLS-fw: right ventricular free wall peak longitudinal strain derived by 3D echocardiography; 3D-RVLS-sw: right ventricular septal wall peak longitudinal strain derived by 3D echocardiography

^a^Wilcoxon signed-rank test

### Observer variability

The ICC for the intra-observer reliability for the RVEDV was 0.924 (95% CI: 0.741–0.980), RVESV 0.847 (95% CI: 0.524–0.959), RVEF 0.984 (95% CI: 0.942–0.996), 3D-RVLS-fw 0.927 (95% CI: 0.749–0.981) and RVFAC 0.669 (95% CI: 0.143–0.905). The ICC for the inter-observer reliability for the RVEDV was 0.927 (95% CI: 0.737–0.981), RVESV 0.957 (95% CI: 0.839–0.989), RVEF 0.846 (95% CI: 0.497–0.959), 3D-RVLS-fw 0.834 (95% CI: 0.466–0.956) and RVFAC 0.662 (95% CI: 0.052–0.09.12).

## Discussion

Anesthesia induction with endotracheal intubation and consecutive positive pressure ventilation constitutes a vulnerable phase for the right ventricle. Increased pulmonary vascular resistance due to the formation of atelectasis, hypercapnia or hypoxia can lead to right ventricular afterload increases. Additionally, positive pressure ventilation is known to alter right ventricular filling pressures. One of the first reports in 1948 showed that intrathoracic pressure was inversely correlated with right ventricular filling pressures [18]. Several subsequent studies have shown that positive pressure ventilation with high PEEP levels can lead to consecutive right heart dysfunction [19–21]. It is supposed that this is due to a reduction in venous return and increases in right ventricular afterload. Contrary to these studies an animal study by Luecke et al. showed that increasing levels of PEEP up to 21 cmH_2_O did not alter RV function [22]. This finding is supported by a small study in cardiac surgery patients that applied the open-lung concept with PEEP levels of 17 cmH_2_O [23].

In addition to the effect of mechanical ventilation, right ventricular function can be altered by hemodynamic changes, as well as the direct negative effects of anesthesia agents on right ventricular myocardial contractility. Propofol, for example, has been shown to alter systemic hemodynamic parameters without influencing the RVEDV or RVEF, measured by pulmonary artery catheter-based techniques [24, 25]. Midazolam, the sedative agent used in this study, is known to have little to no impact on pulmonary vascular resistance 5 min after anesthesia induction, but can reduce both the left- and right-ventricular stroke work indices [26, 27]. The impact of anesthetics on the left ventricular function has been previously investigated. In a study by Bhavsar et al. a bolus of 1.5-2 μg/kg sufentanil for anesthesia induction lead to a significantly decreased left ventricular global longitudinal strain whereas systemic hemodynamic parameters or stroke volume remained unchanged [28]. In a study during anesthesia induction in pediatric cardiac patients, left ventricular ejection fraction decreased after administration of sufentanil or fentanyl [29]. In an investigation by Frederiksen et al., these findings could not be confirmed [30]. The authors showed that sufentanil and remifentanil do not affect left ventricular global longitudinal strain [30]. Studies assessing the impact of opioids on the right ventricle are not available. There are controversial data in the literature regarding the effects of sevoflurane on right ventricular function. While the administration of 1–1.5 MAC of sevoflurane led to a significant decrease in right ventricular stroke work index (RVSWI) in anaesthetized pigs [31], pulmonary vascular resistance increased but RVSWI remained stable in another porcine study using a MAC of less than 1 [32]. Malan et al. studied the effects of sevoflurane on healthy volunteers and showed that systemic vascular resistance and mean pulmonary artery pressures are reduced [33]. Furthermore, they observed neither a change in left sided preload, measured by the left ventricular end-diastolic area, nor in left ventricular fractional area change [33].

To date and to the best of our knowledge, no other study has comprehensively assessed established parameters of right ventricular mechanics and novel 3D echocardiographic parameters of right ventricular function during anesthesia induction and initiation of positive pressure ventilation. Right ventricular volume, quantified by the RVEDVI, and the RVEDAI did not change in our study. This is contrary to previously published results by Couture et al. that showed an increase in preload as measured by the RVEDA after anesthesia induction [34]. PEEP and peak inspiratory pressure levels are not reported in the article of Couture et al. [34], thus making it difficult to compare their findings to our results. A potential reason for the different observations could be the different approach for the volumetric quantification, particularly the direct measurement of the RVEDVI in this study. The 3DE volumetric technique incorporates the whole right ventricle. This technique includes the right ventricular outflow tract in the volume calculation and overcomes the use of a single imaging plane used by Couture et al.. Foreshortened imaging planes in 2D echocardiography could be another potential reason for the different results. In our patients RVEF and stroke volume did not significantly change. This finding is supported by the data published by Pinsky et al. that showed no relevant change in ejection fraction during various PEEP levels [5]. Our data demonstrate that the longitudinal function of the right ventricle, as measured by indices that assess the movement of the lateral tricuspid annulus, namely, TAPSE and TDITVs’, are significantly reduced after anesthesia induction and IPPV. This confirms the previous findings of Tousignant et al. that have shown a similar reduction in TAPSE [35]. But contrary to our results they did not experience a change in TDITVs’ what could be due to the different patient population, the smaller number of included patients or different ventilator settings. The movement of the tricuspid annulus is not solely associated with right ventricular function and relies on the movement of the whole heart. Moreover, TAPSE has shown to be associated with left ventricular function [36, 37]. In addition, conflicting data exist about the lack of correlation of the TAPSE with cardiac MRI-derived RV ejection fraction [38, 39]. Imaging of longitudinal myocardial deformation does not only rely on the movement of the tricuspid annulus and directly assesses the longitudinal myocardial contractility. Right ventricular strain imaging is considered to detect even subtle changes in myocardial contractility [40]. In our patients we observed a significant but small decrease in right ventricular free wall strain. Due to the fact that the right ventricular myocardium has different contraction components, the reduction of the longitudinal contraction could have been compensated by the circumferential, antero-posterior or the twisting component leading to a stable RVEF and SV.

In a subgroup analysis we can show that patients with a normal RVEF at baseline experienced a significant decrease in the longitudinal myocardial contraction component accompanied by a small but significant reduction in RVEF. Interestingly, in the subgroup of patients with depressed RVEF (< 35%) no change of any of the RV parameters could be observed. This suggests that in patients with already reduced RVEF anesthesia and IPPV do not further worsen right ventricular myocardial function. Due to the small sample size of the subset (*n* = 12) additional larger studies in this specific patient collective are needed to confirm or falsify this finding.

In this prospective study, we showed the effects of anesthesia induction and initiation of positive pressure ventilation on right ventricular function. Our data clearly demonstrate that echocardiographic parameters assessing the tricuspid annular movement (TAPSE and TDITVs’), are significantly reduced despite stable loading conditions. The reduction in tricuspid annular movement might be directly associated with changes in intrathoracic pressures by the initiation of positive pressure ventilation. The longitudinal right ventricular myocardial contraction (3D-RVLS-fw) was also reduced in our patient population. In addition we showed that the ejection fraction assessed by 3DE also slightly decreased during anesthesia induction in a subgroup of patients presenting with a normal baseline RVEF but, however, stroke volume did not change.

### Clinical implications

This study shows a decrease in longitudinal right ventricular function after anesthesia induction using sufentanil, midazolam and rocuronium and IPPV with currently recommended ventilator settings. In a group of patients with a normal baseline RVEF the RVEF also slightly decreased but RV stroke volume stayed stable. Despite the reduction in longitudinal function and the slight reduction in RVEF (mean − 3%) this effect seems not to be directly clinically relevant as the stroke volume remained stable. However, echocardiographers need to be aware that longitudinal RV function is depressed under anesthesia and intraoperative values should be cautiously compared to baseline measurements when judging RV function. Modern 3DE techniques especially 3D volumetry improve insights into right heart dimensions and function and enhance our understanding of anesthesia-related changes of ventricular function and physiology. Therefore, 3D echocardiography should be used to complement 2D echocardiographic examinations in the perioperative setting.

### Study limitations

Our data have several limitations. The patients included in our single-center study do not represent a healthy collective but a cross section through patients presenting in cardiac anesthesia with various underlying diseases. Unfortunately, we cannot report on the changes in right ventricular afterload in our patients. The results for the 2D echocardiography measurements of RV longitudinal function could be biased because some values were missing during data collection. Although overall changes are small, the sample size might have been too small to reach significance for some of the 3D-based echocardiography parameters. The assessment of different right ventricular contraction components – such as longitudinal and circumferential shortening - could not be performed but might have given further insights into complex alterations of the myocardial contraction patterns. We do acknowledge that we can only report on the initial changes in right ventricular function and cannot address if this effect is reversible after emergence from anesthesia. In addition, the 3D technology that we used is not yet available in a broad clinical setting.

## Conclusions

Our results suggest that tricuspid annular movement and longitudinal RV function decreases after anesthesia induction and positive pressure ventilation, while the stroke volume does not change. The latter finding questions the clinical relevance of the small changes seen. Further investigations are necessary to confirm this finding, especially in patients with a depressed baseline RVEF and during anesthesia induction with other anesthetics or different ventilator settings.

## Data Availability

The datasets used and/or analyzed during the current study are available from the corresponding author on reasonable request.

## Abbreviations

2D: Two-dimensional
3DE: 3-dimensional echocardiography
3D-RVLS-fw: Right ventricular free wall peak longitudinal strain
3D-RVLS-sw: Right ventricular septal-wall peak longitudinal strain
BMI: Body-mass-index
CMRI: Cardiac magnetic resonance imaging
DBP: Diastolic blood pressure
ICC: Intraclass correlation Coefficients
IPPV: Intermittent positive pressure ventilation
IQR: Interquartile ranges
IVC: Inferior vena cava
MAC: Minimal alveolar concentration
MBP: Mean blood pressure
PEEP: Positive end expiratory pressure
Pinsp: Peak inspiratory pressure
RV: Right ventricle
RVEDA: Right ventricular enddiastolic area
RVEDAI: Right ventricular enddiastolic area indexed to body surface area
RVEDV: Right ventricular end-diastolic volume
RVEDVI: Right ventricular end-diastolic volume indexed to body-surface area
RVEF: Right ventricular ejection fraction
RVESV: Right ventricular end-systolic volume
RVESVI: Right ventricular end-systolic volume indexed to body-surface area
RVESWI: Right ventricular stroke work index
RVFAC: Right ventricular fractional area change
SBP: Systolic blood pressure
SD: Standard deviation
SV: Stroke volume
TAPSE: Tricuspid annular plane systolic excursion
TDITVs’: Tissue Doppler derived systolic movement of the lateral tricuspid annulus
TTE: Transthoracic echocardiography
